# Targeted delivery of liposomal chemoimmunotherapy for cancer treatment

**DOI:** 10.3389/fimmu.2022.1010021

**Published:** 2022-10-19

**Authors:** Yusheng Liu, Joonsu Han, Yang Bo, Rimsha Bhatta, Hua Wang

**Affiliations:** ^1^ Department of Materials Science and Engineering, University of Illinois at Urbana-Champaign, Urbana, IL, United States; ^2^ Cancer Center at Illinois (CCIL), Urbana, IL, United States; ^3^ Department of Bioengineering, University of Illinois at Urbana-Champaign, Urbana, IL, United States; ^4^ Carle College of Medicine, University of Illinois at Urbana-Champaign, Urbana, IL, United States; ^5^ Beckman Institute for Advanced Science and Technology, University of Illinois at Urbana-Champaign, Urbana, IL, United States; ^6^ Materials Research Laboratory, University of Illinois at Urbana-Champaign, Urbana, IL, United States; ^7^ Institute for Genomic Biology, University of Illinois at Urbana-Champaign, Urbana, IL, United States

**Keywords:** immunotherapy, chemo-immunotherapy, metabolic glycan labeling, liposome, click chemistry

## Abstract

Chemoimmunotherapy that utilizes the immunomodulatory effect of chemotherapeutics has shown great promise for treating poorly immunogenic solid tumors. However, there remains a significant room for improving the synergy between chemotherapy and immunotherapy, including the efficient, concurrent delivery of chemotherapeutics and immunomodulators into tumors. Here, we report the use of metabolic glycan labeling to facilitate cancer-targeted delivery of liposomal chemoimmunotherapy. 4T1 triple-negative breast cancer cells can be metabolically labeled with azido groups for subsequently targeted conjugation of dibenzocycoloctyne (DBCO)-bearing liposomes loaded with doxorubicin and imiquimod (R837) adjuvant *via* efficient click chemistry. The encased doxorubicin can induce the immunogenic death of cancer cells and upregulate the expression of CD47 and calreticulin on the surface of cancer cells, while R837 can activate dendritic cells for enhanced processing and presentation of tumor antigens. Targeted delivery of liposomes encapsulating doxorubicin and R837 to 4T1 tumors, enabled by metabolic glycan labeling and click chemistry, showed the promise to reshape the immunosuppressive tumor microenvironment of solid tumors. This cancer-targetable liposomal chemoimmunotherapy could provide a new approach to improving conventional chemotherapy.

## Introduction

The past decades have witnessed significant progress in the clinical treatment of cancers, especially with the success of cancer immunotherapy including checkpoint blockades, chimeric antigen receptor (CAR) T-cell therapy, and cancer vaccines (1–3). However, the limited patient response rate, poor efficacy against many solid tumors, and occasionally severe side effects still limit the utility of immunotherapy (3–5). Currently, the vast majority of cancer patients are still suffering from conventional chemotherapy and radiation therapy with limited efficacy and severe side effects. As some chemotherapeutic agents can induce the immunogenic death of cancer cells and thus activate the immune system to assist in tumor clearance (6–8), strategies to integrate chemotherapy with immunomodulatory agents have also been extensively explored. For example, doxorubicin (Dox), a first-line chemotherapeutics for treating breast cancer and other cancers, can upregulate the immunogenic markers of tumor cells and facilitate the release of tumor antigens from dying tumor cells, which has been utilized for the development of combination therapies with checkpoint blockades or adjuvants (9–11). Despite recent progress, the optimization of the synergy between chemotherapeutics and immunomodulators, upon a better understanding of the immunomodulatory effect within the tumor microenvironment, is still needed (12–14). Ideally, tumor antigens released from dying tumor cells as a result of chemotherapy can be fully exploited by antigen-presenting cells (e.g., dendritic cells (DCs)) for subsequent priming of antigen-specific effector T cells, as a part of the mechanism to reshape the immunosuppressive tumor microenvironment towards favoring immune cell infiltration and tumor killing.

Concurrent delivery of chemotherapeutics that function towards tumor cells and immunomodulatory agents that modulate either DCs or T cells to the tumorous tissues remains another challenge (15–18). Targeted delivery of these molecules to the tumorous tissues could minimize off-target side effects. However, the separate modulation of tumor cells and immune cells by chemotherapeutics and immunomodulators within the tumor microenvironment limits the choice of suitable tumor-targeting technologies (19–21). For strategies that target the tumor matrix (22–25), drug delivery systems can release co-loaded chemotherapeutics and immunomodulators in the tumor matrix, which then function on tumor and immune cells, respectively. For strategies that target tumor cells (26–28), we envision the use of nonmetabolizable immunomodulators would be necessitated for the optimal synergy with chemotherapeutics. The internalized immunomodulators can then be released from the dying tumor cells for subsequent modulation of immune cells in the tumor microenvironment. For example, doxorubicin (Dox) can induce the apoptosis and immunogenic death of tumor cells and eventually facilitate the release of cellular constituents from dying tumor cells. By co-delivering Dox and R837 (29–31), a nonmetabolizable adjuvant for activating DCs (also known as Imiquimod), into tumor cells, R837 can be released into the tumor microenvironment upon the death of tumor cells, activate DCs, and facilitate the processing and presentation of tumor antigens by DCs, thereby amplifying antitumor T-cell responses.

Here, we report the use of metabolic glycan labeling and click chemistry to achieve targeted delivery of liposomes co-encapsulating chemotherapeutics and adjuvants to tumor cells and tumorous tissues. Unnatural sugars such as tetraacetyl-*N*-azidoacetylmannosamine (Ac_4_ManNAz) can undergo the metabolic glycoengineering processes and become expressed on the cell membrane in the form of glycoproteins and glycolipids (32–36). The cell-surface azido groups can then mediate targeted conjugation of dibenzocyclooctyne (DBCO)-bearing agents *via* efficient and bioorthogonal click chemistry (37–41). Metabolic glycan labeling coupled with click chemistry has been successfully utilized for cancer-targeted delivery of therapeutics, diagnostics, and nanoparticles (42, 43). Upon covalent conjugation, agents can be gradually internalized by cells *via* the endocytic pathway. In this study, we aim to target liposomes co-encapsulating Dox and R837, a nonmetabolizable adjuvant for activating DCs, to tumor cells. After covalent conjugation and cellular internalization, Dox is expected to induce the apoptosis of cancer cells, while the nonmetabolizable R837 remains intact intracellularly and eventually becomes released by the dead cancer cells. We show that liposomes co-encapsulating Dox and R837 can induce the immunogenic death of cancer cells and activation of DCs, and with simple DBCO modification, can be efficiently conjugated to azido-labeled cancer cells. *In vivo* metabolic labeling of tumor cells with azido groups, followed by administration of DBCO-modified Dox/R837-loaded liposomes, also manages to reshape the tumor microenvironment by activating DCs, repolarizing tumor-associated macrophages, and improving the tumor infiltration of T cells.

## Results and discussions

**Figure 1:**
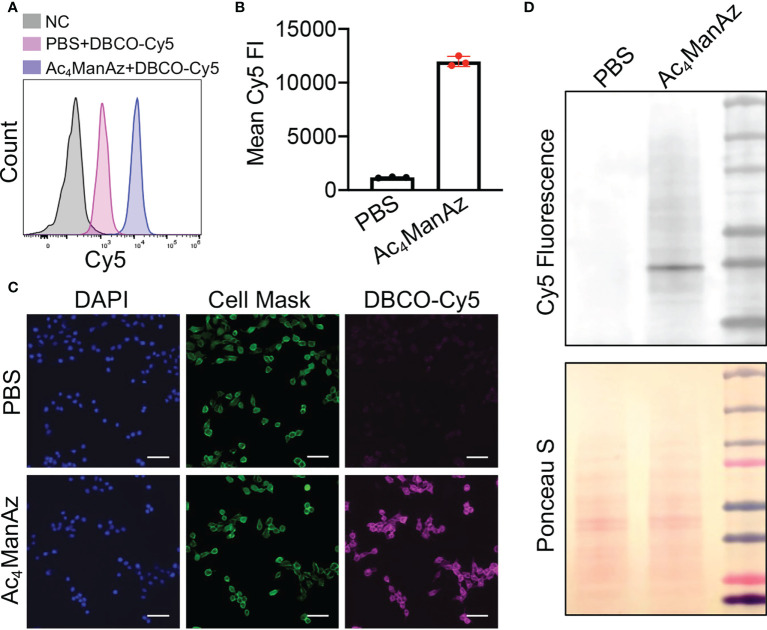
Metabolic glycan labeling of 4T1 cancer cells with azido groups. 4T1 cells were incubated with Ac_4_ManNAz (25 μM) or PBS for 48 h, and then stained with DBCO-Cy5 for detection of cell-surface azido groups. **(A)** Representative Cy5 histograms of 4T1 cells for different groups. 4T1 cells without any treatment were used as negative controls. **(B)** Mean Cy5 fluorescence intensity of 4T1 cells pretreated with Ac_4_ManNAz (25 μM) and PBS, respectively. **(C)** Fluorescence images of Ac_4_ManNAz- or PBS-pretreated 4T1 cells. Cell nuclei and membranes were stained with DAPI and Cell Mask, respectively. Scar bar: 50 μm. **(D)** Western blot analysis of azido-labeled glycoproteins. Azido-labeled proteins were conjugated with DyLight™ 650-Phosphine for fluorescence visualization. Ponceau S stains of whole protein bands were also shown.

We first studied whether tetraacetyl-*N*-azidoacetylmannosamine (Ac_4_ManNAz) can metabolically label 4T1 breast cancer cells with azido groups. After treating with Ac_4_ManNAz for 48 h, cells were incubated with DBCO-Cy5 for the detection of cell-surface azido groups. Flow cytometry analysis showed much higher Cy5 fluorescence intensity of Ac_4_ManNAz-treated 4T1 cells than untreated cells (**Figures 1A, B**; **Supplementary Figure S1**), indicating the successful metabolic labeling of 4T1 cells with azido groups. The Ac_4_ManNAz-mediated metabolic labeling efficiency of 4T1 cells was concentration-dependent (**Supplementary Figure S2A**). Higher concentrations of Ac_4_ManNAz (>25 µM) could not only result in enhanced metabolic labeling efficiency but also exhibited noticeable cytotoxicity (**Supplementary Figure S2B**). For these reasons, 25 µM Ac_4_ManNAz was used in all subsequent *in vitro* experiments. Confocal imaging confirmed the significantly higher Cy5 fluorescence intensity of Ac_4_ManNAz-treated cells than control cells and showed a good overlay between Cy5 signal and membrane stain (**Figure 1C**). To validate whether azido groups were expressed in the form of glycoproteins, we also performed a Western blot analysis of 4T1 cell lysates and detected the azido-tagged glycoproteins using DyLight™ 650-Phosphine, which can be conjugated with azido groups. As a result, Ac_4_ManNAz-treated 4T1 cells, unlike control cells, showed various protein bands under the Cy5 fluorescence channel (**Figure 1D**), demonstrating the successful azido labeling of cell-surface glycoproteins.

**Figure 2:**
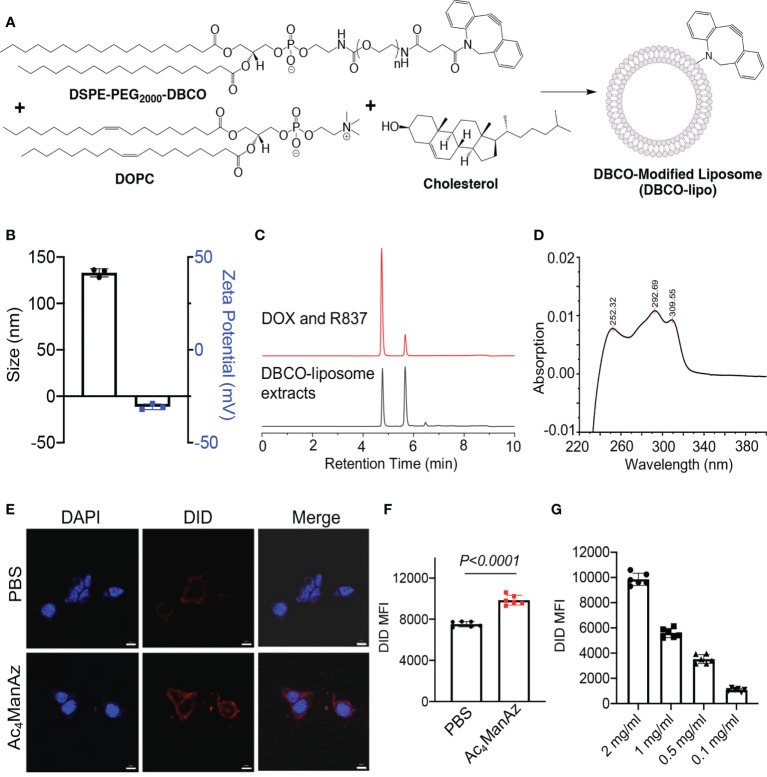
Synthesis, characterization, and *in vitro* targeting effect of DBCO-modified liposomes. **(A)** Schematic illustration of the synthesis of DBCO-modified liposomes from DSPE-PEG_2000_-DBCO, DOPC, and cholesterol. Unmodified liposomes were synthesized using DSPE-PEG_2000_. **(B)** Size and zeta potential of DBCO-lipo. **(C)** HPLC traces of a mixture of Dox and R837 or the extracts of Dox/R837-encapsulating DBCO-lipo. The detection wavelength was set at 254 nm. **(D)** The UV absorption spectrum of DBCO-lipid as detected at ~6.5 min in **(C)**. **(E)** Confocal images of 4T1 cells pretreated with Ac_4_ManNAz or PBS for 48 h and then incubated with DID-encapsulating DBCO-lipo for 30 min. Scale bar: 10 μm. **(F)** Mean Cy5 fluorescence intensity of 4T1 cells pretreated with Ac_4_ManNAz or PBS for 48 h and then incubated with DID-encapsulating DBCO-lipo (2 mg/ml) for 30 min. **(G)** Mean Cy5 fluorescence intensity of 4T1 cells pretreated with Ac_4_ManNAz and then incubated with different concentrations of DID-encapsulating DBCO-lipo for 30 min. All the numerical data are presented as mean ± SD.

We next synthesized DBCO-modified liposomes using DOPC, cholesterol, and DSPE-PEG_2000_-DBCO at a molar ratio of 6:4:0.5, following the reported membrane extrusion method (**Figure 2A**). Control liposomes without DBCO modification were synthesized similarly by replacing DSPE-PEG_2000_-DBCO with DSPE-PEG_2000_. The as-synthesized DBCO-liposomes have an average diameter of 133 nm and a zeta potential of −11.3 mV (**Figure 2B**; **Supplementary Figure S3**), similar to that of control liposomes (**Supplementary Figure S4A**). Dox and R837 can be easily encapsulated into the liposomes during the lipid film hydration step, with unloaded Dox and R837 removed *via* size exclusion chromatography (**Supplementary Figure S4B**). The loaded Dox and R837 can be quantified *via* HPLC, and a loading of 25 µg/mg Dox and 12.5 µg/mg R837 can be easily achieved (**Supplementary Figures S5A, B**). To validate the successful incorporation of DBCO functionality, we extracted liposomal components with methanol and analyzed the extracts *via* HPLC. At a detection absorption wavelength of 254 nm, where the nonaromatic structures would be invisible, the extracts of DBCO-modified, Dox/R837-loaded liposomes showed three main peaks (**Figure 2C**). The first two peaks corresponded to Dox and R837, respectively, while the third peak at ~6.5 min corresponded to DBCO-lipid (**Figure 2C**), which was further confirmed by the absorption spectrum (**Figure 2D**). Prior to the *in vitro* cell targeting study, we also fabricated DBCO-modified, DID-loaded liposomes, using a similar encapsulation strategy to Dox and R837, to enable fluorescence tracking. 4T1 cells pretreated with Ac_4_ManNAz or PBS for 48 h were incubated with DBCO-modified, DID-loaded liposomes for 30 min and imaged under a confocal microscope. Compared to the control cells with azido-sugar treatment, 4T1 cells pretreated with Ac_4_ManNAz showed a significantly higher DID fluorescence signal (**Figure 2E**). Flow cytometry analysis also confirmed the higher DID signal in Ac_4_ManNAz-pretreated 4T1 cells than in control cells (**Figure 2F**). The intracellular DID signal also increased with the concentration of DBCO-modified, DID-loaded liposomes (**Figure 2G**). These experiments demonstrated that azido-labeled cancer cells can mediate targeted conjugation of DBCO-modified liposomes *via* efficient click chemistry.

**Figure 3:**
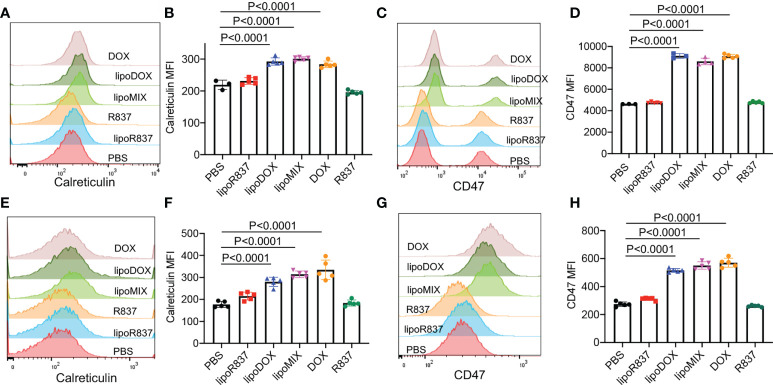
Dox-loaded liposomes can induce the immunogenic death of cancer cells. 4T1 breast cancer cells **(A–D)** or B16F10 melanoma **(E–H)** were incubated with free Dox, Dox-loaded liposomes (lipoDox), Dox/R837-loaded liposomes (lipoMix), R837-loaded liposomes (lipoR837), free R837, or PBS for 16 h, followed by staining with anti-CD47 and anti-calreticulin prior to flow cytometry analysis. The concentration of Dox and R837 was set at 500 and 330 nM, respectively. **(A)** Representative calreticulin histograms and **(B)** mean calreticulin fluorescence intensity of 4T1 cells after different treatments. Also shown are **(C)** representative CD47 histograms and **(D)** mean CD47 fluorescence intensity of 4T1 cells after different treatments. **(E)** Representative calreticulin histograms and **(F)** mean calreticulin fluorescence intensity of B16F10 cells after different treatments. Also shown are **(G)** representative CD47 histograms and **(H)** mean CD47 fluorescence intensity of B16F10 cells after different treatments. All the numerical data are presented as mean ± SD.

**Figure 4:**
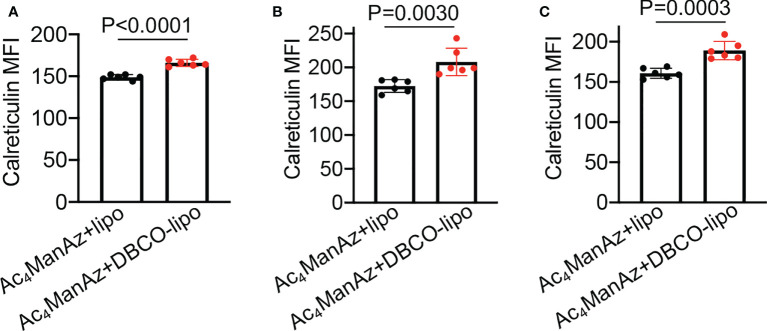
*In vitro* targeting of DBCO-liposomes to azido-labeled 4T1 cancer cells improves immunogenic cell death. 4T1 cells were pretreated with Ac_4_ManNAz for 48 h, followed by incubation with DBCO-modified or DBCO-unmodified, Dox-loaded liposomes at different Dox concentrations for 16 h. Shown are the mean calreticulin fluorescence intensity of 4T1 cells after treatment with liposomes at a Dox concentration of **(A)** 50 nM, **(B)** 500 nM, or **(C)** 5 µM. All the numerical data are presented as mean ± SD.

After demonstrating the *in vitro* targeting effect of DBCO-liposomes on azido-labeled cancer cells, we next fabricated Dox/R837-loaded liposomes (lipoMix), Dox-loaded liposomes (lipoDox), and R837-loaded liposomes (lipoR837), respectively, and studied their ability to induce the immunogenic death of 4T1 breast cancer cells and B16F10 melanoma. Compared to control cells, 4T1 cells treated with Dox, lipoMix, or lipoDox for 16 h showed an upregulated expression of cell-surface calreticulin (**Figures 3A, B**), which has been reported as a biomarker for the immunogenic death of cancer cells (42–47). Compared to free Dox, lipoMix and lipoDox resulted in a comparable surface expression of calreticulin (**Figures 3A, B**). In contrast, 4T1 cells treated with R837 or lipoR837 caused a negligible change in the surface expression of calreticulin (**Figures 3A, B**), ruling out the effect of R837 on the immunogenic death of cancer cells. In addition to calreticulin, 4T1 cells treated with Dox, lipoMix, or lipoDox also upregulated the surface expression of the antiphagocytic marker CD47 compared to control cells (**Figures 3C, D**), which was previously demonstrated to aid in immunoevasion by cancer cells. R837 alone did not induce an increased expression of CD47 (**Figures 3C, D**) (10, 45–48). We also examined the surface expression of CD47 and calreticulin by 4T1 cells after treatment with Dox or LipoDox for 0, 2, 8, 16, 24, and 48 h, respectively. The expression levels of CD47 and calreticulin increased from 2 to 16 h and stayed stable after 16 h (**Supplementary Figures S6A, B**). It is noteworthy that 48-h incubation with Dox or LipoDox resulted in a significant reduction in 4T1 viability (**Supplementary Figure S6C**). A similar phenomenon was observed for B16F10 cells, i.e., an upregulated surface expression of calreticulin (**Figures 3E, F**) and CD47 (**Figures 3G, H**) by B16F10 cells treated with Dox, lipoMix, or lipoDox in comparison with control cells. These experiments demonstrated that Dox and Dox-loaded liposomes can induce the immunogenic death of cancer cells. We also studied whether *in vitro* targeting of lipoDox to cancer cells can improve immunogenic cancer cell death. 4T1 cells were pretreated with Ac_4_ManNAz to express azido groups on the cell surface and then incubated with DBCO-lipoDox or lipoDox for 16 h. At all tested concentrations (50 nM, 500 nM, or 5 µM in Dox equivalents), DBCO-lipoDox induced a significantly higher expression of calreticulin on 4T1 cancer cells in comparison with lipoDox (**Figures 4A–C**).

**Figure 5:**
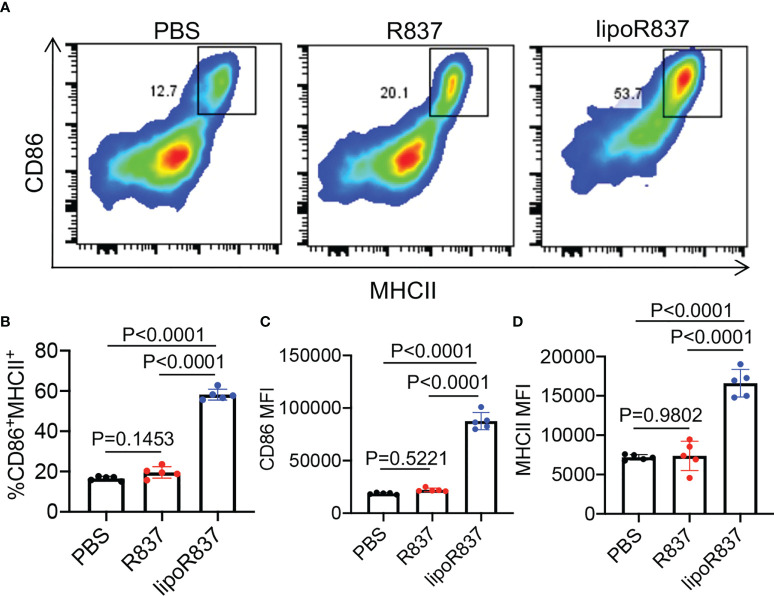
R837-loaded liposome outperforms R837 in improving the activation of DCs. BMDCs were incubated with free R837, R837-loaded liposomes (lipoR837), or PBS for 16 h, followed by FACS analysis of cell-surface CD86 and MHCII levels. **(A)** Representative CD86-MHCII plots of DCs after 16-h treatment with R837, lipoR837, or PBS. **(B)** Percentages of CD86^+^MHCII^+^ DCs following different treatments. Also shown are the mean **(C)** CD86 and **(D)** MHCII fluorescence intensity of DCs after different treatments. All the numerical data are presented as mean ± SD.

**Figure 6:**
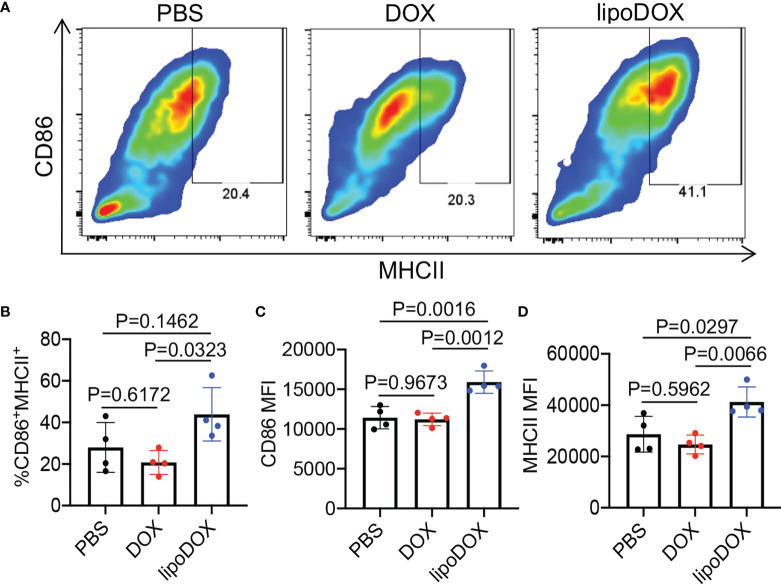
4T1 cancer cells treated with Dox-loaded liposomes can induce the activation of DCs. 4T1 cells were treated with free Dox, Dox-loaded liposomes (lipoDox), or PBS for 16 h, and then co-cultured with BMDCs for another 16 h. **(A)** Representative CD86-MHCII plots of DCs after 16-h co-culture with 4T1 cells. **(B)** Percentages of CD86^+^MHCII^+^ DCs following different treatments. Also shown are the mean **(C)** CD86 and **(D)** MHCII fluorescence intensity of DCs after different treatments. All the numerical data are presented as mean ± SD.

After demonstrating the capability of Dox-loaded liposomes to induce the immunogenic death of cancer cells, we next studied whether R837-loaded liposomes can mediate the activation of DCs. Bone marrow-derived DCs (BMDCs) were incubated with free R837, R837-loaded liposomes (lipoR837), or PBS for 16 h, followed by FACS analysis of CD86 and MHCII expression levels (**Figure 5A**). LipoR837 resulted in significantly improved activation of DCs in comparison with free R837, as evidenced by a higher percentage of CD86^+^MHCII^+^ DCs (**Figure 5B**), a higher expression level of CD86 (**Figure 5C**), and a higher expression level of MHCII (**Figure 5D**). These experiments demonstrated that lipoR837 outperforms free R837 in activating DCs, presumably as a result of the enhanced accumulation of lipoR837 in endosomes where TLRs 7/8 (receptors for R837) exist. To better understand the crosstalk of cancer cells and DCs, we also studied whether Dox-treated cancer cells can improve the activation of DCs. 4T1 cells were treated with lipoDox, free Dox, or PBS for 24 h, followed by the removal of drug-containing cell media and the addition of BMDCs. After 16-h incubation, the levels of cell-surface CD86 and MHCII on BMDCs were analyzed *via* flow cytometry (**Figure 6A**). Compared to 4T1 cells pretreated with free Dox, cells pretreated with lipoDox resulted in a significantly higher expression level of CD86 and MHCII on DCs during the co-culture (**Figures 6B–D**), demonstrating the superior ability of lipoDox to induce the apoptosis and immunogenic death of cancer cells for subsequent activation of DCs.

**Figure 7:**
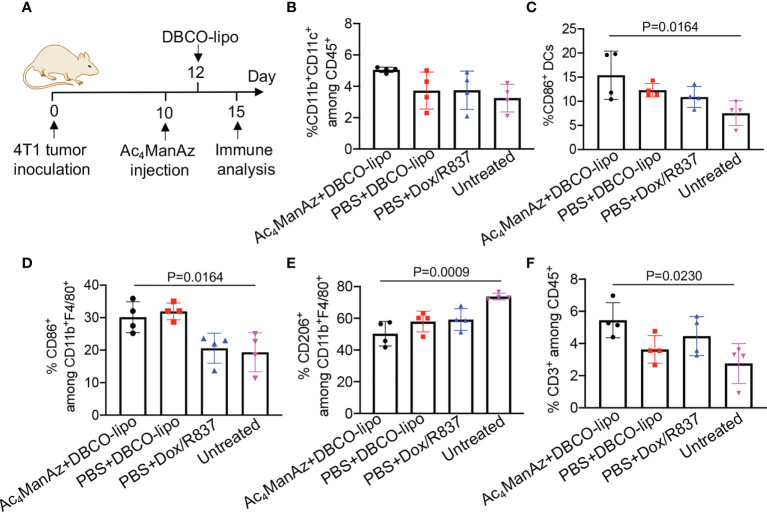
Azido sugar coupled with Dox/R837-loaded DBCO-liposomes alters the immunosuppressive tumor microenvironment. **(A–F)** 4T1 tumors were inoculated on day 0, followed by intratumoral injection of Ac_4_ManNAz (or PBS) on day 10 and DBCO-liposomes (or a mixture of Dox and R837) on day 12. On day 15, tumors were collected for immune analysis. **(A)** Timeframe of tumor study. Shown are the percentages of **(B)** CD11b^+^CD11c^+^ DCs among CD45^+^ immune cells, **(C)** CD86^+^ cells among CD11b^+^CD11c^+^ DCs, **(D)** CD86^+^ cells among CD11b^+^F4/80^+^ macrophages, **(E)** CD206^+^ cells among CD11b^+^F4/80^+^ macrophages, and **(F)** CD3^+^ T cells among CD45^+^ immune cells in tumors after different treatments. All the numerical data are presented as mean ± SD.

After demonstrating the ability of Dox/R837-loaded liposomes to induce the immunogenic death of cancer cells and activation of DCs *in vitro*, we next studied whether targeting Dox/R837-loaded liposomes to 4T1 tumors has the potential to reshape the immunosuppressive tumor microenvironment. Balb/c mice bearing established 4T1 tumors were intratumorally injected with Ac_4_ManNAz or PBS, followed by injection of DBCO-modified, Dox/R837-loaded liposomes or a mixture of Dox/R837 48 h later. After 3 days, tumors were harvested for immune analysis (**Figure 7A**). Compared to the untreated group, azido-labeling coupled with DBCO-liposome was able to increase the population of CD11b^+^CD11c^+^ DCs in 4T1 tumors (**Figure 7B**). Among the CD11b^+^CD11c^+^ DCs, a higher fraction of the CD86^+^ population was also observed in tumors labeled with azido groups and then treated with DBCO-liposomes, in comparison with untreated mice (**Figure 7C**). The targeting group also managed to increase the fraction of CD86^+^ M1-phenotype macrophages (**Figure 7D**), decrease the fraction of CD206^+^ M2-phenotype macrophages (**Figure 7E**), and increase the fraction of CD3^+^ T cells (**Figure 7F**) in tumors compared to the untreated group. Compared to mice pretreated with PBS and then DBCO-liposomes, the targeting group resulted in a slight increase in the fractions of DCs, CD86^+^ DCs, and CD3^+^ T cells, and a slight decrease in CD206^+^ M2-phenotype macrophages (**Figures 7B–F**), which we expect to further improve upon the optimization of the dose and dosing frequency of azido sugars and DBCO-liposomes.

## Materials and methods

d-Mannosamine hydrochloride and other chemicals were purchased from Sigma-Aldrich (St. Louis, MO, USA) unless otherwise noted. DBCO-Cy5 was purchased from Thermo Fisher Scientific (Waltham, MA, USA). GM-CSF was purchased from PeproTech (Cranbury, NJ, USA). Primary antibodies used in this study, including PE-conjugated anti-CD11b (Invitrogen), PE/Cy7-conjugated anti-CD11c (Invitrogen), APC-conjugated anti-CD86 (Invitrogen), PE/Cy5.5-conjugated anti-CD3-ϵ (Invitrogen), Alexa Fluor 700-conjugated anti-CD8-α (Invitrogen), PE/Cy7-conjugated anti-PD-1 (Invitrogen), PE-conjugated anti-CTLA-4 (Invitrogen), APC-conjugated anti-LAG-3 (Invitrogen), and Alexa Fluor 700-conjugated anti-MHCII (Invitrogen) were purchased from Thermo Fisher Scientific (Waltham, MA, USA). Fixable viability dye efluor780 was obtained from Thermo Fisher Scientific (Waltham, MA, USA). All antibodies were diluted according to the manufacturer’s recommendations. Small molecule compounds were run on the Agilent 1290/6140 ultra-high-performance liquid chromatography/mass spectrometer or the Shimadzu high-performance liquid chromatography. Proton and carbon nuclear magnetic resonance spectra were collected on the Varian U500 or VXR500 (500 MHz) spectrometer. Fluorescent images were taken with a EVOS microscope (Thermofisher, Waltham, MA, USA). Fluorescence measurement was conducted on a Biotek plate reader (BioTek Instruments, Winooski, VT, USA). FACS analyses were collected on Attune NxT flow cytometers and analyzed on Flowjo™ 10. Statistical testing was performed using GraphPad Prism v8.

### Cell lines and animals

The 4T1 cell line was purchased from the American Type Culture Collection (Manassas, VA, USA). Cells were cultured in RPMI 1640 containing 10% FBS and 100 units/ml penicillin/streptomycin at 37°C in 5% CO_2_-humidified air. Female Balb/c mice were purchased from the Jackson Laboratory (Bar Harbor, ME, USA). Feed and water were available ad libitum. Artificial light was provided in a 12 h/12 h cycle. All procedures involving animals were done in compliance with the National Institutes of Health and Institutional guidelines with approval from the Institutional Animal Care and Use Committee at the University of Illinois at Urbana-Champaign.

### Methods

#### Synthesis of Ac_4_ManNAz

Mannosamine hydrochloride (2.3 mmol, 1.0 e.q.) was suspended in methanol, followed by the addition of sodium methoxide in methanol (25%, w/w) (2.3 mmol, 1.0 e.q.). The mixture was stirred until the mannosamine hydrochloride was dissolved. Triethylamine (1.1 e.q.) was then added to the solution upon vigorous stirring, followed by the addition of chloroacetic anhydride (1.1 e.q.). The reaction mixture was stirred overnight. Sodium azide (4.0 e.q.) was then added, and the reaction mixture was stirred at 65°C for another 12 h. Upon removal of the solvent under reduced pressure, pyridine was added to dissolve the residue. Acetic anhydride and a catalytic amount of DMAP were then slowly added to the mixture on a 0°C bath, with the mixture stirred overnight. Water was added to quench the reaction and the solvent was removed on a rotavapor. The crude product was purified *via* silica gel column chromatography using ethyl acetate as the eluent (60% overall yield). ^1^H NMR (CDCl_3_, 500 MHz): δ (ppm) 6.66 and 6.60 (d, *J* = 9.0 Hz, 1H, C(O)N*H*CH), 6.04 and 6.04 (d, 1H, *J* = 1.9 Hz, NHCHC*H*O), 5.32–5.35 and 5.04–5.07 (dd, *J* = 10.2, 4.2 Hz, 1H, CH_2_CHC*H*CH), 5.22 and 5.16 (t, *J* = 9.9 Hz, 1H, CH_2_CHCHC*H*), 4.60–4.63 and 4.71–4.74 (m, 1H, NHC*H*CHO), 4.10–4.27 (m, 2H, C*H*_2_CHCHCH), 4.07 (m, 2H, C(O)C*H*_2_N_3_), 3.80–4.04 (m, 1H, CH_2_C*H*CHCH), and 2.00–2.18 (s, 12H, C*H*_3_C(O)). ^13^C NMR (CDCl_3_, 500 MHz): δ (ppm) 170.7, 170.4, 170.3, 169.8, 168.6, 168.3, 167.5, 166.9, 91.5, 90.5, 73.6, 71.7, 70.5, 69.1, 65.3, 65.1, 62.0, 61.9, 52.8, 52.6, 49.9, 49.5, 21.1, 21.0, 21.0, 20.9, 20.9, 20.9, and 20.8. ESI MS (*m/z*): calculated for C_16_H_22_N_4_O_10_Na [M+Na]^+^ 453.1, found 453.1.

#### Synthesis of DBCO-liposomes

Different lipids dissolved in the chloroform (10 mg/ml) were added to the glass vial with a composition of DOPC:CHOL:DSPE-PEG_2000_-DBCO = 6:4:0.5 (molar ratio). DSPE-PEG_2000_-DBCO was replaced with DSPE-mPEG_2000_ to synthesize the control liposome. The solvent was removed on a rotary evaporator to form a thin lipid film on the vial wall. The vial was further freeze-dried overnight to completely remove the solvent. PBS was added to the vial to yield a 10-mg/ml of lipid solution, which was vortexed for 30 min at room temperature to hydrate the lipid film. After water bath sonication for 15 min, the resulting liposomes were further purified *via* a membrane extrusion method by passing through a 100-nm membrane eight times with the Avanti extruder.

#### Loading of R837 and DOX into liposomes

DOX and R837 were encapsulated into the liposomes during the hydration process. Briefly, an equal volume of DOX (2 mg/ml in H_2_O) and R837 (2 mg/ml in pH = 2 HCl a.q. solution) were mixed to yield a mixture of 1 mg/ml of DOX and 1 mg/ml of R837. The mixture was added to dry lipid film, followed by 30-min vortex at room temperature to hydrate the lipid film. The lipid solution was then sonicated on a water bath for 15 min and extruded through a 100-nm membrane eight times to yield liposomes. To remove the unencapsulated DOX and R837, liposomes were further purified by passing through a size exclusion column with PBS as the eluent. The encapsulated DOX and R837 were quantified with HPLC. The size and zeta potential of liposomes were characterized by a Malvern dynamic light scattering (DLS).

#### Synthesis of DID-labeled DBCO-liposome

DID was encapsulated into the liposomes during the lipid film formation step. DID in methanol was added to the lipid mixture, followed by the removal of solvents on a rotary evaporator to form a lipid film. The lipid film was further freeze-dried overnight and hydrated with PBS. The lipid solution was then sonicated on a water bath for 15 min and extruded through a 100-nm membrane eight times to yield liposomes.

#### *In vitro* metabolic glycan labeling and flow cytometry detection

4T1 cancer cells were seeded in a six-well plate with a density of 2 × 10^5^ cells per well, and Ac_4_ManNAz was added with a final concentration of 25 μM. A 50-mM stock solution of Ac_4_ManNAz in DMSO was diluted with PBS to yield different concentrations. After incubation for 48 h, cells were washed with PBS three times, followed by incubation with 50 μM DBCO-Cy5 in Opti-MEM for 30 min. Cells were then detached from the plate with trypsin, washed with FACS buffer twice, and resuspended in FACS buffer containing 1% paraformaldehyde, prior to flow cytometry analysis.

#### Fluorescence imaging of metabolically labeled cells

4T1 cells (2 × 10^4^ cells per well) in 1 ml of DMEM were seeded into a six-well plate with glass coverslips, and Ac_4_ManNAz was added with a final concentration of 25 μM. After incubation for 48 h, cells were washed with PBS three times, followed by incubation with 50 μM DBCO-Cy5 in Opti-MEM for 30 min. After washing, cells were stained with the Membrane Green and DAPI following the manuals, fixed with 4% paraformaldehyde, and imaged under a fluorescence microscope.

### Western blot analysis of azido-labeled cells

4T1 cells (2 × 10^5^ cells per well) in 5 ml of medium was seeded into a culture dish in the presence or absence of 25 μM AAM and cultured for 48 h. After washing with PBS, cells were lifted with trypsin and harvested. To each cell pellet, ice-cold cell lysis buffer (1% SDS, 100 mM Tris·HCl, pH 7.4) with 1× protein inhibitor cocktail was added, and the sample was sonicated for 5 min on an ice bath. Sonicated cell lysates were placed at 4°C for 30 min to further dissolve the proteins. The cell debris was removed by centrifugation with 21,000×*g* at 4°C for 10 min. The protein concentration in the supernatant was determined by bicinchoninic acid (BCA) protein assay (Pierce, IL, USA) and was adjusted to 2 mg/ml. To 10 μl of the solution, 0.5 μl DyLight™ 650-Phosphine (2 mM in DMSO) was added. The mixture was placed at 37°C overnight; 2× SDS-PAGE loading buffer was then added, and 10 μl of each sample was loaded to 4%–12% SDS-PAGE gels after heating at 95°C for 5 min. After SDS-PAGE gel electrophoresis, protein bands were transferred to the nitrocellulose membrane. The membrane was imaged on a Quan800 under the Cy5 channel. Protein bands were also confirmed by the Ponceau S staining.

#### Flow cytometry analysis of DBCO-liposome conjugation to azido-labeled 4T1 cells

4T1 cells (2 × 10^4^ cells per well) are seeded to a six-well plate, followed by the addition of Ac_4_ManNAz with a final concentration of 25 μM. After incubation for 48 h, cells were washed with HBSS and incubated with different concentrations (2, 1, or 0.5 mg/ml) of DID-loaded DBCO-liposomes for 30 min at 37°C. After washing with PBS three times, cells were treated with 0.25% trypsin and harvested for flow cytometry analysis.

#### Immunogenic death of 4T1 and B16F10 cells *in vitro*

4T1 or B16F10 cells were seeded in a 96-well plate with a density of 2 × 10^4^ cells per well and allowed to attach overnight. Cells were then incubated with free Dox, free R837, Dox-loaded liposomes, R837-loaded liposomes, Dox/R837-loaded liposomes, or PBS for 16 h. The concentrations of Dox were set at 50, 500, or 5,000 nM. The concentrations of R837 were set at 33, 330, or 3,300 nM. The supernatant was removed and cells were lifted by treating with 0.25% trypsin. After washing with cold FACS buffer twice, cells were incubated with anti-CD47-FITC and Rabbit anticalreticulin primary antibody at 4°C for 30 min. After washing, Goat-anti-Rabbit-Cy5 secondary antibody and Fixable Viability Dye eFluor™ 780 were added to stain cells at 4°C for 30 min. Cells were then fixed with 0.4% PFA solution and analyzed on a flow cytometer.

#### *In vitro* BMDC activation

BMDCs were isolated from the tibia and femur of C57BL/6 mice following the previously reported method. Cells on day 6 or 7 were used. BMDCs were seeded to a 96-well plate with a density of 4 × 10^4^ cells per well, and different forms (free or liposome) and concentrations (high, medium, or low) of Dox and R837 were added. After 16 h, cells were stained with APC-conjugated anti-CD11c, PE-conjugated anti-CD86, Alexa Fluor 700-conjugated anti-MHCII, and Fixable Viability Dye eFluor™ 780 for 30 min at 4°C. Cells were then washed, fixed with 0.4% PFA, and resuspended in FACS buffer for flow cytometry analysis.

#### Cocultures of 4T1 cells and BMDCs

4T1 cells (2 × 10^4^ cells per well) were seeded to a 96-well plate and incubated with different forms (free or liposome) and concentrations (high, medium, or low) of DOX and R837 for 16 h. The supernatant was removed and cells were washed with HBSS. BMDCs (4 × 10^4^ cells per well) were added to each well and cocultured with 4T1 cells for another 16 h. BMDCs were harvested, washed, and stained with PE-Cy7-conjugated anti-CD11c, PE-conjugated anti-CD86, FITC-conjugated anti-MHCII, and Fixable Viability Dye eFluor™ 780 at 4°C for 30 min. After washing, cells were resuspended with 0.4% PFA and analyzed on a flow cytometer.

#### Tumor study

Balb/c mice (6–8 weeks) were subcutaneously inoculated with 4T1 tumor cells (1 × 10^6^ cells in 50 µl HBSS) on day 0. On day 10 when the tumors grow to a size of ~100 mm^3^, mice were randomly divided into four groups: Ac_4_ManNAz + DBCO-liposome, PBS + DBCO-liposomes, a mixture of Dox and R837, or PBS. Ac_4_ManNAz (20 µl, 430 µg) or PBS was intratumorally injected. After 48 h, 20 µl of DBCO-liposome loaded with Dox (1 mg/ml) and R837 (0.5 mg/ml) or a bolus mixture of DOX and R837 was intratumorally injected. Three days later, tumors were collected, disrupted, digested, and resuspended in FACS buffer. Cells were then stained with the antibody cocktail at 4°C for 30 min, fixed with 0.4% PFA, and analyzed on a flow cytometer.

## Conclusion

To summarize, we report the use of metabolic glycan labeling and click chemistry for targeted delivery of liposomal chemoimmunotherapy to cancer cells. Azido sugars (e.g., Ac_4_ManNAz) can metabolically label cancer cells with azido groups, for subsequently targeted conjugation of DBCO-bearing agents, including DBCO-modified liposomes, *via* efficient click chemistry. We show that liposomes co-encapsulating Dox and R837 were able to induce the immunogenic death of 4T1 and B16F10 cancer cells and the activation of DCs. Ac_4_ManNAz-mediated metabolic labeling, coupled with DBCO-modified Dox/R837-loaded liposomes, was able to improve the immunogenic cancer death and activation of DCs *in vitro* and showed the promise to reshape the immunosuppressive tumor microenvironment *in vivo*. Further optimization of the dose and dosing frequency of azido sugars and DBCO-liposomes will enable the improved synergy between Dox and R837. Our strategy of targeting liposomal chemoimmunotherapy to tumors could provide an effective approach to improving conventional chemotherapy.

### Statistical analyses

Statistical analysis was performed using GraphPad Prism v6 and v8. Sample variance was tested using the *F* test. For samples with equal variance, the significance between the groups was analyzed by a two-tailed student’s *t*-test. For samples with unequal variance, a two-tailed Welch’s *t*-test was performed. For multiple comparisons, a one-way analysis of variance (ANOVA) with *post-hoc* Fisher’s LSD test was used. The results were deemed significant at 0.01 < ^*^ *p* ≤ 0.05, highly significant at 0.001 < ^**^ *p* ≤ 0.01, and extremely significant at ^***^ *p* ≤ 0.001.

## Data availability statement

The raw data supporting the conclusions of this article will be made available by the authors, without undue reservation.

## Ethics statement

The animal study was reviewed and approved by IACUC of UIUC.

## Author contributions

YL and HW conceived the project and designed the experiments. YL, JH, YB and RB performed the experiments. YL, JH, Y, RB and HW analyzed the data. YL and HW wrote the manuscript. All authors contributed to the article and approved the submitted version.

## Funding

The authors would like to acknowledge the financial support from NSF DMR 21-43673 CAR, R01CA274738, and the start-up package from the Department of Materials Science and Engineering at the University of Illinois at Urbana-Champaign and the Cancer Center at Illinois. Research reported in this publication was supported by the Cancer Scholars for Translational and Applied Research (C*STAR) Program sponsored by the Cancer Center at Illinois and the Carle Cancer Center under Award Number CST EP012023.

## Conflict of interest

The authors declare that the research was conducted in the absence of any commercial or financial relationships that could be construed as a potential conflict of interest.

## Publisher’s note

All claims expressed in this article are solely those of the authors and do not necessarily represent those of their affiliated organizations, or those of the publisher, the editors and the reviewers. Any product that may be evaluated in this article, or claim that may be made by its manufacturer, is not guaranteed or endorsed by the publisher.
