# Supplementary material for: Targeted delivery of liposomal chemoimmunotherapy for cancer treatment
Source: Front Immunol. 2022 Oct 19;13:1010021. doi: 10.3389/fimmu.2022.1010021 (PMC9626969; doi:10.3389/fimmu.2022.1010021)
Supplement: Supplementary file 1 [file DataSheet_1.pdf]

Targeted Delivery of Liposomal Chemo-Immunotherapy for Cancer Treatment

Yusheng Liu[1], Joonsu Han[1], Yang Bo[1], Rimsha Bhatta[1], Hua Wang[1,2,3,4,5,6,7*]

[1]Department of Materials Science and Engineering, University of Illinois at Urbana-Champaign, Urbana, IL 61801, USA. [2]Cancer Center at Illinois (CCIL), Urbana, IL 61801, USA. [3]Department of Bioengineering, University of Illinois at Urbana-Champaign, Urbana, IL 61801, USA. [4]Carle College of Medicine, University of Illinois at Urbana-Champaign, Urbana, IL 61801, USA. [5]Beckman Institute for Advanced Science and Technology, University of Illinois at Urbana-Champaign, Urbana, IL 61801, USA. [6]Materials Research Laboratory, University of Illinois at Urbana-Champaign, Urbana, IL 61801, USA. [7]Institute for Genomic Biology, University of Illinois at Urbana-Champaign, Urbana, IL 61801, USA.

*correspondence should be addressed to huawang3@illinois.edu

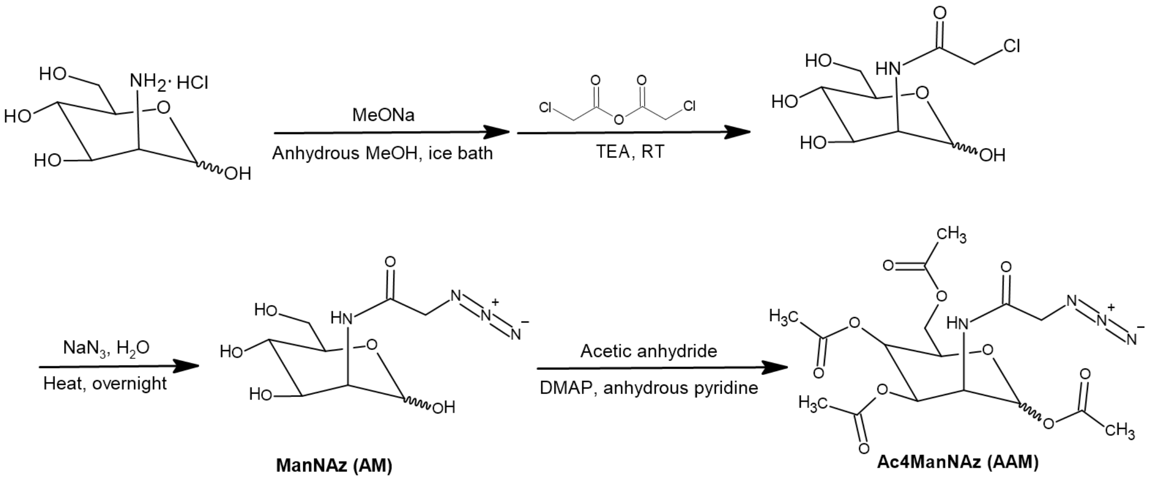

Figure S1. Synthetic route of $Ac_4ManNAz$

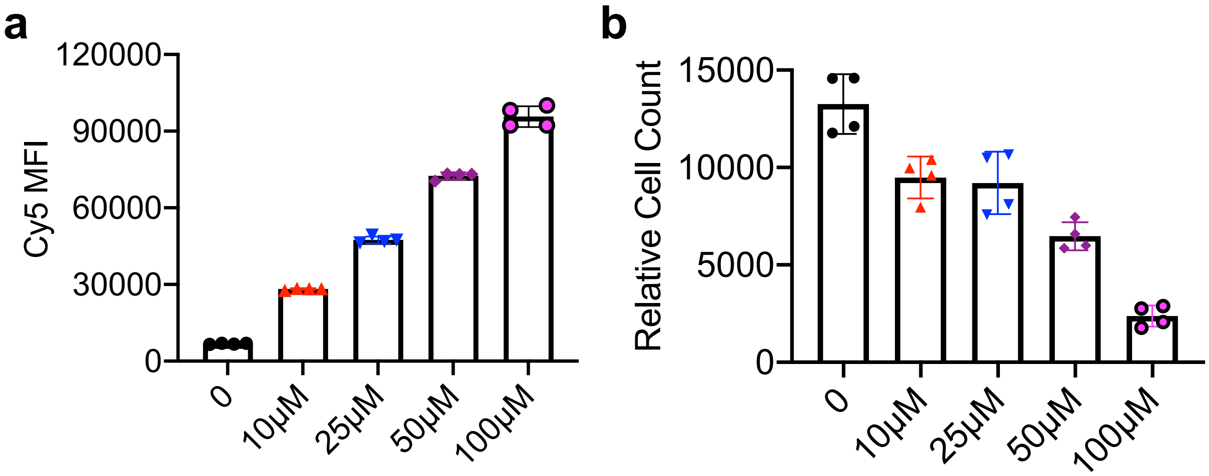

Figure S2. (a) Mean Cy5 fluorescence intensity of 4T1 cells pretreated with different concentrations of Ac_4ManAz for 48 h and incubated with DBCO-Cy5 for 30 min. (b) Relative cell counts of 4T1 cells pretreated with different concentrations of Ac_4ManAz for 48 h.

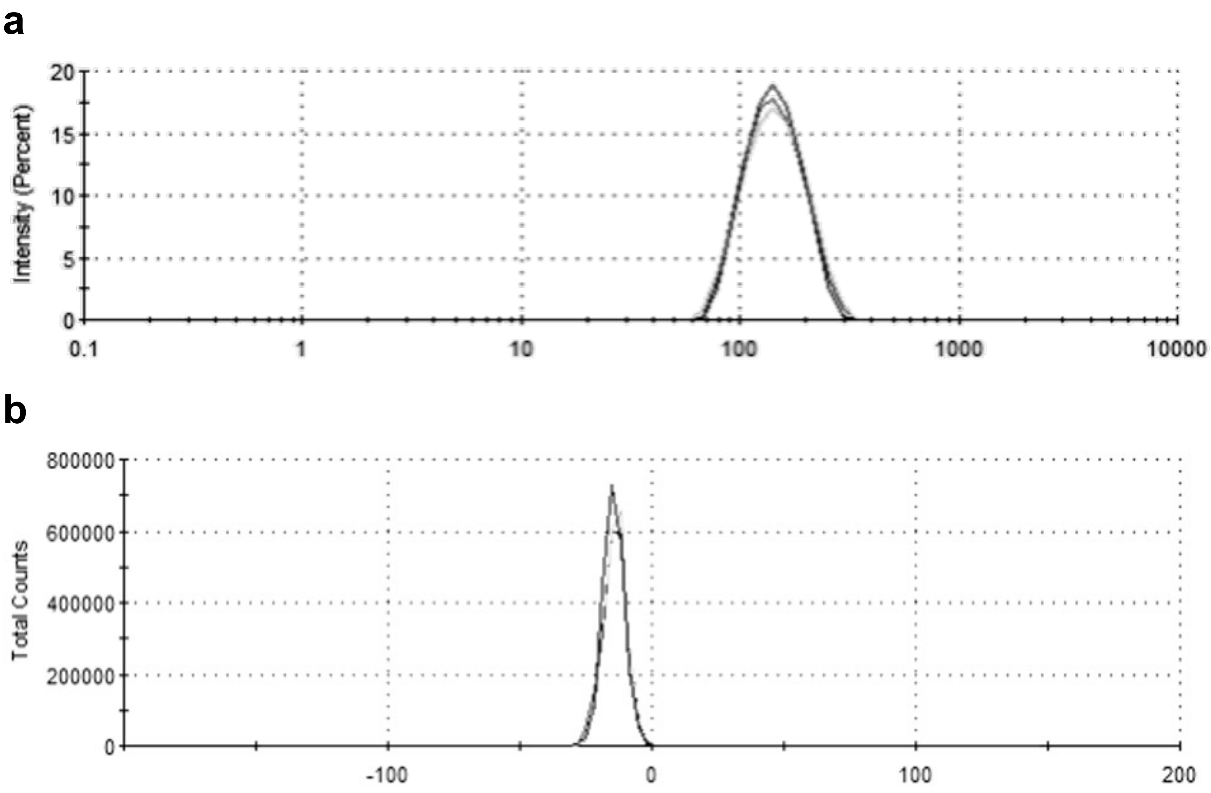

Figure S3. (a) Size distribution and (b) zeta potential of DBCO-liposomes.

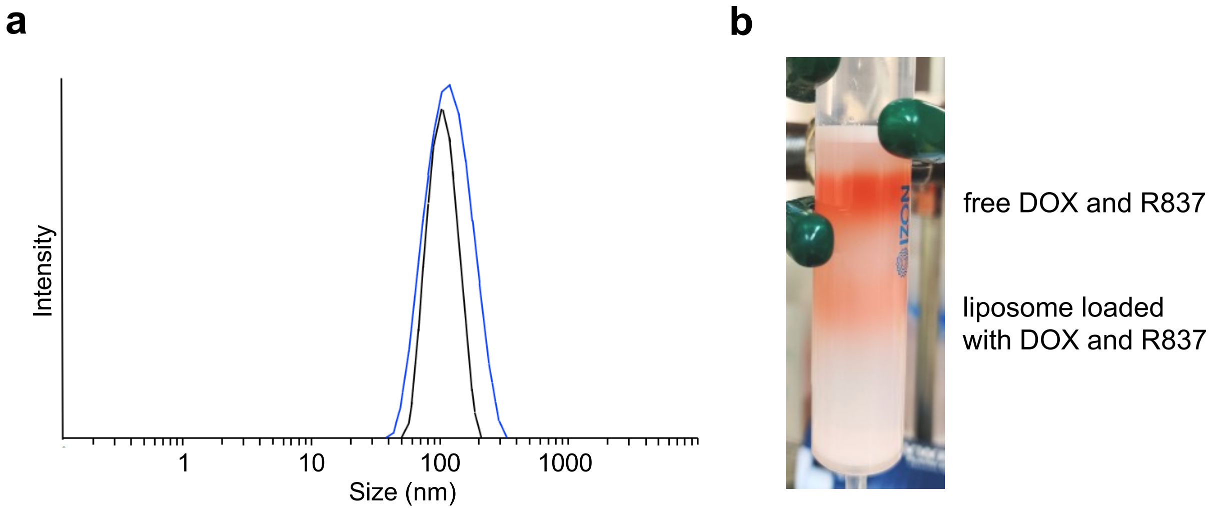

Figure S4. (a) Size distribution of DBCO-liposomes (blue) and unmodified liposomes (black). (b) Purification of Dox/R837-loaded liposomes via a size exclusion column.

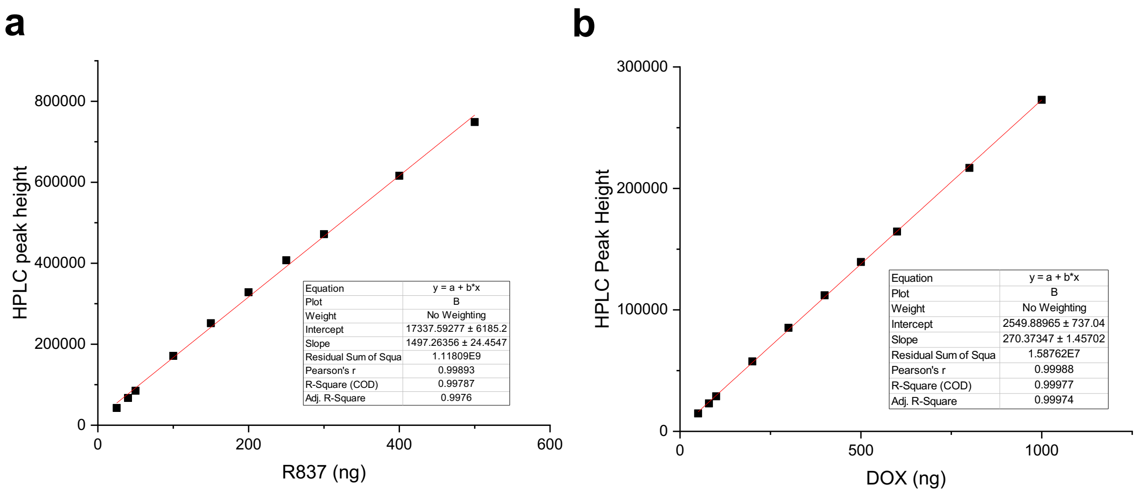

Figure S5. Standard curves for quantifying the loading of (a) R837 and (b) Dox in liposomes. Detection wavelength was set at 254 nm.

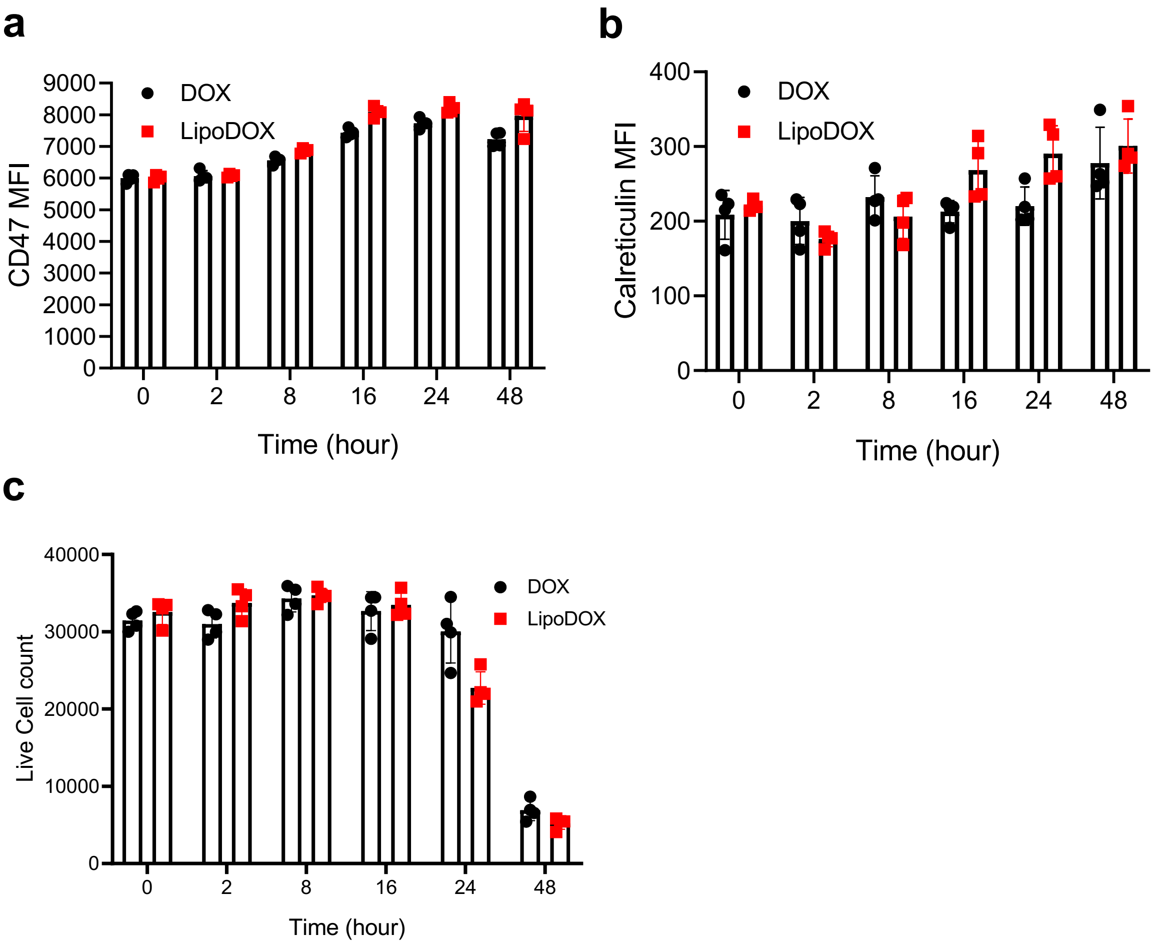

Figure S6. (a) CD47 expression levels of 4T1 cells after treatment with Dox or lipoDox (500 nM in Dox equivalents) for varied times. (b) Cell-surface calreticulin levels of 4T1 cells after treatment with Dox or lipoDox (500 nM in Dox equivalents) for varied times. (c) Relative cell viability of 4T1 cells after treatment with Dox or lipoDox (500 nM in Dox equivalents) for varied times.

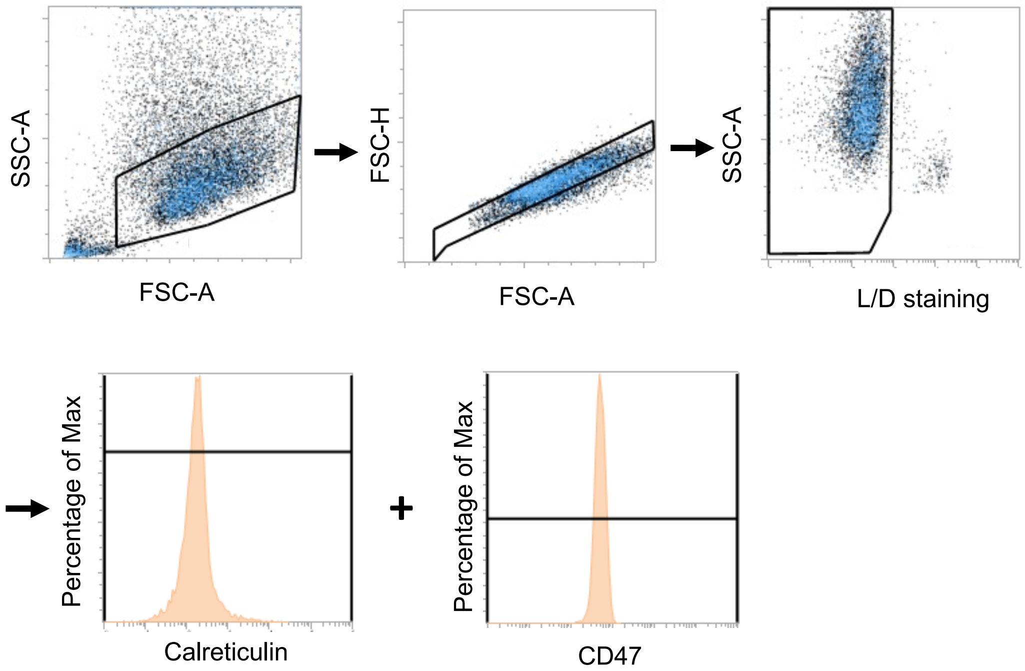

Figure S7. Representative gating strategy for analyzing immunogenic death of cancer cells.

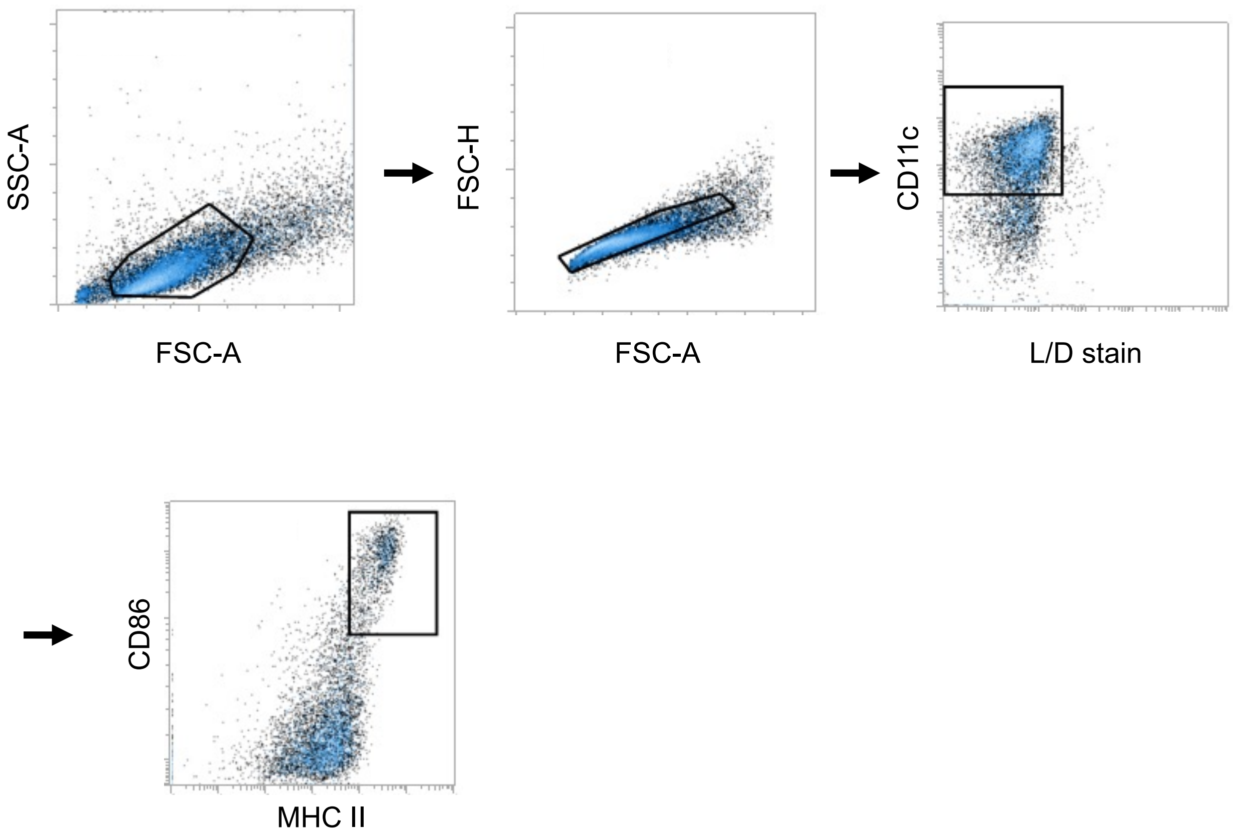

Figure S8. Representative gating strategy for analyzing activation status of DCs.

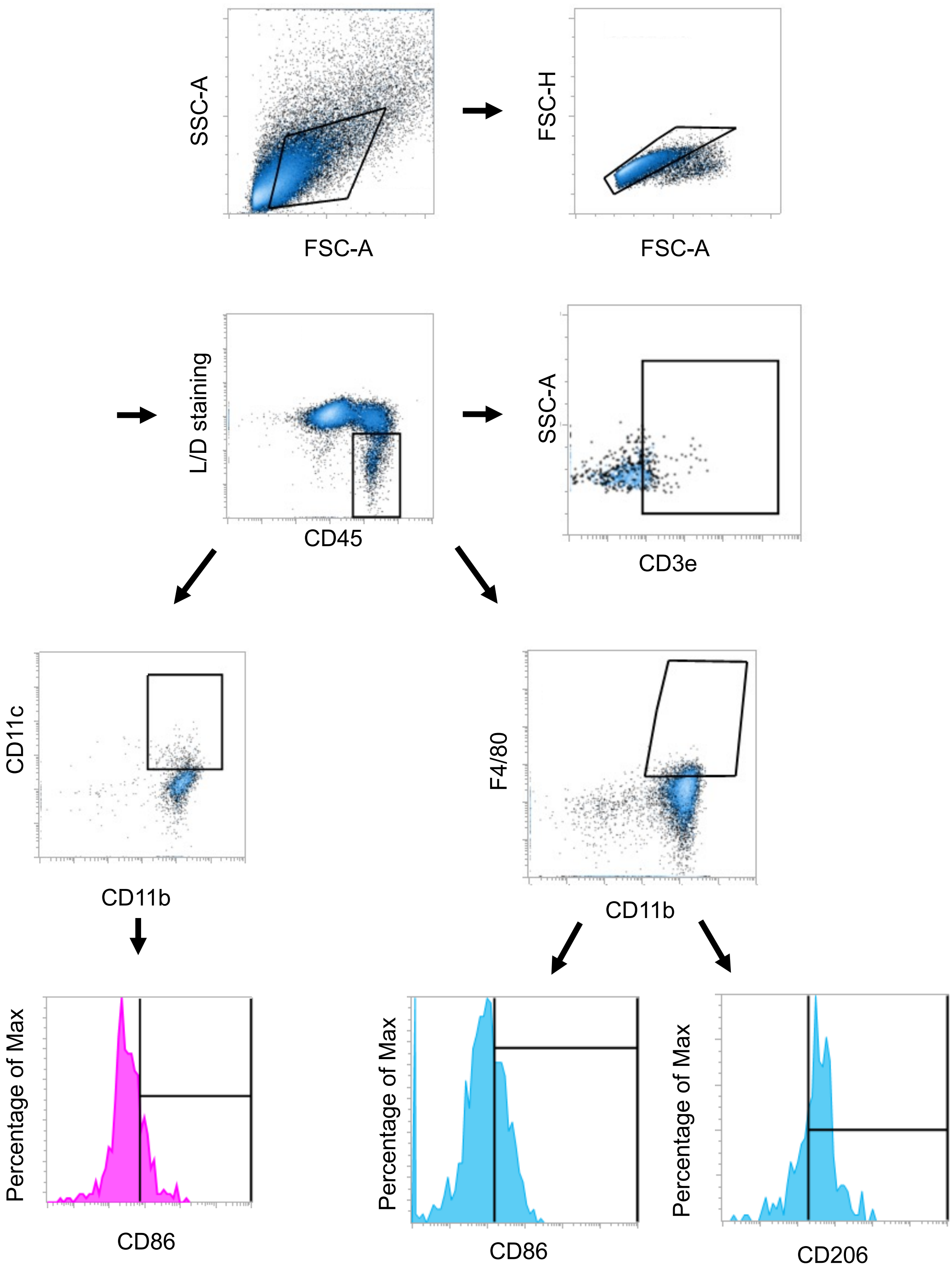

Figure S9. Representative gating strategy for immune analysis of tumors.
